# Low‐intensity pulsed ultrasound attenuates cardiac inflammation of CVB3‐induced viral myocarditis via regulation of caveolin‐1 and MAPK pathways

**DOI:** 10.1111/jcmm.14098

**Published:** 2018-12-27

**Authors:** Cheng Zheng, Sen‐Min Wu, Hao Lian, Yuan‐Zheng Lin, Rong Zhuang, Saroj Thapa, Quan‐Zhi Chen, Yi‐Fan Chen, Jia‐Feng Lin

**Affiliations:** ^1^ Department of Cardiology The Second Affiliated Hospital and Yuying Children's Hospital of Wenzhou Medical University Wenzhou China; ^2^ Department of Ultrasound The Second Affiliated Hospital and Yuying Children's Hospital of Wenzhou Medical University Wenzhou China; ^3^ Department of Intensive Care Unit The Second Affiliated Hospital and Yuying Children's Hospital of Wenzhou Medical University Wenzhou China; ^4^ The Second School of Medicine of Wenzhou Medical University Wenzhou China

**Keywords:** augmented inflammatory response, caveolin‐1, ERK, low‐intensity pulsed ultrasound, p38 MAPK, viral myocarditis

## Abstract

The aggressive immunological activity elicited by acute viral myocarditis contributes to a large amount of cardiomyocytes loss and poor prognosis of patients in clinic. Low‐intensity pulsed ultrasound (LIPUS), which is an effective treatment modality for osteoarthropathy, has been recently illustrated regulating the overactive inflammatory response in various diseases. Here, we aimed to investigate whether LIPUS could attenuate coxsackievirus B3 (CVB3) infection‐induced injury by coordinating the inflammatory response. Male BALB/c mice were inoculated intraperitoneally with CVB3 to establish the model of acute viral myocarditis. LIPUS treatment was given on Day 1, Day 1, 3 and Day 1, 3, 5 post‐inoculation, respectively. All mice were followed up for 14 days. Day 1, 3, 5 LIPUS treatment significantly improved the survival rate, attenuated the ventricular dysfunction and ameliorated the cardiac histopathological injury of CVB3‐infected mice. Western blotting analysis showed Day 1, 3, 5 LIPUS treatment decreased pro‐inflammatory cytokines, increased the activation of caveolin‐1 and suppressed p38 mitogen‐activated protein kinase (MAPK) and extracellular signal‐regulated kinase (ERK) signallings in heart tissue. RAW264.7 cells were treated with lipopolysaccharides (LPS) to simulate the augmented inflammatory response in vivo. LIPUS treatment on RAW264.7 inhibited the expression of pro‐inflammatory cytokines, activated caveolin‐1 and suppressed p38 MAPK and ERK signallings. Transfecting RAW264.7 with caveolin‐1 siRNA blunted the suppression of pro‐inflammatory cytokines and MAPK signallings by LIPUS treatment. Taken together, we demonstrated for the first time that LIPUS treatment attenuated the aggressive inflammatory response during acute viral myocarditis. The underlying mechanism may be activating caveolin‐1 and suppressing MAPK signallings.

## 1 INTRODUCTION

Viral infection‐induced myocarditis is one of the leading causes of acute heart failure and malignant ventricular arrhythmias among young patients. During the acute phase of the viral myocarditis, the virus invasion not only induced a direct viral cytopathic effect on cardiomyocytes, but also elicited the activation of host immune response.^1^ It has been demonstrated that it was the immune‐mediated indirect myocardial injury but not virus‐mediated direct destroy that resulted in a large amount of myocardium loss, contributing to the acute heart failure and even sudden death in clinic. Therefore, regulation of the aggressive immunological response during this acute phase could be potential and effective therapy for viral myocarditis.^2^, ^3^, ^4^, ^5^

Low‐intensity pulsed ultrasound (LIPUS) was a form of mechanical stimulation delivered via a special device and is widely applied as a treatment modality for osteoarthritis in clinical settings.^6^, ^7^ During past decades, emerging evidences revealed that LIPUS treatment could reduce the expression of pro‐inflammtory cytokines, limit the infiltration of inflammatory cells and modulate the phenotype of inflammatory cells in a series of bone and joint diseases, and consequently accelerate the recovery from diseases.^8^, ^9^, ^10^, ^11^ Moreover, recent animal experiments demonstrated that LIPUS could also benefit several cardiovascular diseases, such as left ventricular remodelling induced by chronic myocardial ischaemia and transverse aortic constriction (TAC), where mechanical stimulation of caveolin‐1 and regulation of its downstream intracellular signallings were critically involved.^12^, ^13^, ^14^ However, so far the effect of LIPUS on acute viral myocarditis was never investigated. Based on the studies mentioned above, we doubted whether LIPUS treatment, which had an inflammatory modulating effect, could be utilized as a therapeutic method for the aggressive cardiac inflammation induced by CVB3 infection.

Caveolin‐1, a major component of the plasma membrane microdomains, has been identified in cellular mechanotransduction system sensing the stimulation of LIPUS treatment. It was well known that caveolin‐1 was expressed in various immune cells, including macrophages, dendritic cells and lymphocytes and acted as a potent immunomodulatory molecule via regulating the mitogen‐activated protein kinases (MAPK) family members.^15^, ^16^, ^17^, ^18^

In this study, we aimed to investigate two unknown entities, whether the LIPUS treatment could regulate the aggressive immunological response of CVB3‐induced myocarditis, and if so, to elucidate the underlying molecular mechanisms involved in the beneficial effect.

## 2 MATERIALS AND METHODS

### 2.1 Animal preparation

Male BALB/c mice (4 weeks) were purchased from Shanghai SLAC Laboratory Animal Co., Ltd, China. All mice were kept under the specific pathogen‐free conditions (24 ± 1°C, 45 ± 10% humidity) with a 12‐hour light/dark cycle daily and food and water available ad libitum in the Wenzhou Medical University animal facilities. All animal experiments were approved by the Animal Ethics Committee of Wenzhou Medical University (number wydw2014‐0058) and conformed to the Guide for the Care and Use of Laboratory Animals by the National Institutes of Health.

### 2.2 Myocarditis animal model

CVB3 was amplified by Hep2 cell and harvested. A 50% tissue culture infectious dose (TCID50) assay was used to determine the viral titer. At the age of 5 weeks old, mice were intraperitoneally injected with 0.2 mL phosphate buffered saline (PBS) containing 105 TCID50 CVB3 to induce viral myocarditis. Control mice were intraperitoneally injected with same dosage of PBS. We defined the day of virus inoculation as day 0.

### 2.3 Low‐intensity pulsed ultrasound therapy

The LIPUS therapy was delivered by an ultrasound device (Sonopuls 190; Enraf‐Nonius BV, Rotterdam, the Netherlands) in accordance with the manufacturer's instructions. The parameters of LIPUS therapy adopted for both in vivo and in vitro experiments were based on the specific settings of the device and our preliminary study (Data S1), which were ultrasound frequency of 1 MHz, duty cycle of 20%, pulse repetition frequency of 100 Hz, output intensity of 0.5 W cm^−2^, giving an intensity of spatial average and temporal average of 100 mW cm^−2^. The beams were irradiated from the probe to the mice though an agar phantom gel. LIPUS was applied to the heart in the parasternal short‐axis view at papillary muscle levels with the direction of transthoracic echocardiography for 20 minutes a day under inhalation anaesthesia with 1.2% isoflurane. No‐LIPUS group underwent the same procedures including anaesthesia but without the LIPUS delivered by ultrasound device. In vitro experiments, LIPUS treatment was applied to the bottom of the cultured platelet via an agar phantom gel for 20 minutes.

### 2.4 Group

All control mice were then assigned to two groups blindly, one group was given LIPUS device without ultrasound delivery on day 1, day 3, day 5 (Control group) and another group was given LIPUS device with low‐intensity pulsed ultrasound delivery on day 1, day 3, day 5 (LIPUS group). All CVB3 infected mice was blindly assigned into four groups, the following treatment were given respectively: (a) VMC group: CVB3‐infected mice were given LIPUS device without ultrasound delivery on day 1, day 3, day 5; (b) VMC + D1 LIPUS group: infected mice were given LIPUS device with ultrasound delivery on day 1 and without ultrasound delivery on day 3 and day 5; (c) VMC + D1, 3 LIPUS group: infected mice were given LIPUS device with ultrasound delivery on day 1, day 3 and without ultrasound delivery on day 5; (d) VMC + D1, 3, 5 LIPUS: infected mice were given LIPUS device with ultrasound delivery on day 1, day 3 and day 5. Six mice were selected randomly from each group and killed on days 7 and 14, the hearts of mice were obtained for histological and biochemical examinations, in addition, the blood of mice were collected and plasma level of Troponin I were measured. Preceding to sacrifice, all mice were anaesthetized with pentobarbital (100 mg kg^−1^, administered intraperitoneally). Efficient anaesthesia was evaluated by pinching the hind paw, when sufficient sedation was achieved, the mice were killed by cervical dislocation.

### 2.5 Survival rate

Forty mice extracted from each group were used to monitor survival rate. The survival of each group was observed up to 14 days.

### 2.6 Echocardiographic evaluation

Transthoracic echocardiography was performed with vevo1100 cardiovascular research ultrasound machine (Visualsonics, Japan) on day 14 post‐virus inoculation to evaluate left ventricular function. Mice were anaesthetized by inhalation of isoflurane (1%‐1.5%) prior to echocardiographic evaluation. At the papillary muscle level, the LV end‐diastolic diameter (LVEDd) and LV end‐systolic diameter (LVESd) were measured by long‐axis views of M‐mode tracings from the anterior to posterior left ventricular wall. The left ventricular ejection fraction (LVEF) was further calculated based on LVEDd and LVESd. All measurements were performed by an experienced technician who was blinded to the study groups.

### 2.7 Heart weight (HW), body weight (BW) and HW/BW

The heart weight (HW), body weight (BW) and the ratio of HW to BW (HW/BW) were calculated on day 7 and day 14.

### 2.8 Enzyme‐linked immunosorbent assay (ELISA) measurement of Troponin I in plasma

The blood samples were centrifuged by 1000 *g* for 10 minutes at room temperature, plasma collected was then analysed by a mouse‐specific ELISA kit for Troponin I (Elabscience, E‐EL‐M0086c), following the manufacturer's instruction.

### 2.9 Haematoxylin and eosin (HE) stain

Heart tissues were fixed in 10% formaldehyde, embedded in paraffin, sectioned into 5‐μm‐thick slices and stained with HE. The cardiac inflammatory infiltration was evaluated by observers who were blinded to the experimental groups. Pathological scores were given based on the following criteria: 0 = no lesion; 1 = lesion involving 25% of the myocardium; 2 = lesions involving 25% to 50% of the myocardium; 3 = lesions involving 50% to 75% of the myocardium and 4 = lesions involving 75% to 100% of the myocardium.

### 2.10 Real‐time polymerase chain reaction (PCR)

According to the manufacturer's protocol, total RNA was extracted from the heart tissue with the TRIzol Reagent (Invitrogen Corporation) and converted into cDNA with a RevertAid RT Reverse Transcription Kit (Thermo fisher). Real‐time PCR was performed using SYBR Green I Master (Roche) by LightCycler^®^ 480 System (Roche). The primer sequences applied in the procedure were shown as follows: CVB3: F‐GTCTGCCTGCGTTTATTTC, R‐ACTCAGCGTATCGTTTGGA 510 and GAPDH: F‐AGGGAAATCGTGCGTGACAT, R‐CATCTGCTGGAAGGTGGACA. The relative gene expressions were analysed by the formula 2^−▵▵^CT method.

### 2.11 Western blotting

The proteins were extracted from heart tissues and RAW264.7 cells. Each sample was separated by SDS‐PAGE and transferred to a polyvinylidene difluoride (PVDF) membrane. The membrane was blocked in 5% nonfat dry milk. After being incubated with specific primary antibodies at 4°C overnight and secondary HRP‐conjugated antibodies at room temperature for 1 hour, the membrane was detected with the enhanced chemiluminescence detection system (Millipore, Billerica, MA). The antibodies were applied in our study including: rabbit anti‐Phospho‐caveolin‐1 antibody (dilution 1:1000, Cell signalling technology, #3251), rabbit anti‐Phospho‐p38 MAPK antibody (dilution 1:1000; Cell signalling technology, #4511), rabbit anti‐p38 MAPK antibody (dilution 1:1000; Cell signalling technology, #8690), rabbit anti‐Phospho‐p44/42 MAPK (Erk1/2) antibody (dilution 1:1000; Cell signalling technology, #4370), rabbit anti‐p44/42 MAPK (Erk1/2) antibody (dilution 1:1000; Cell signalling technology, #4695), mouse anti‐IL‐6 antibody (dilution 1:1000; Abcam, ab9324), rabbit anti‐GAPDH antibody (dilution 1:1000; Abcam, ab181602), rabbit anti‐IFN‐gamma antibody (dilution 1:1000; Abcam, ab9918), rabbit anti‐IL‐1β antibody (dilution 1:1000; Bioworld, BS6067), mouse anti‐TNF alpha antibody(dilution 1:1000; Proteintech, 60291), rabbit anti‐caveolin‐1 antibody(dilution 1:1000; Proteintech, 16447‐1‐AP), rabbit anti‐ICAM antibody (dilution 1:1000; Proteintech, 10020), mouse anti‐MCP‐1 antibody (dilution 1:1000; Proteintech, 25542), HRP conjugated goat anti‐mouse secondary antibody(dilution 1:2000; biosharp, BL001A), HRP conjugated goat anti‐rabbit secondary antibody (dilution 1:2000; biosharp, BL003A).

### 2.12 Immunofluorescence

Heart tissues were embedded in the optimal cutting temperature (OCT) and sectioned at a thickness of 5 μm. The sections were then stained with anti‐CD68 primary antibody [dilution 1:500; Proteintech, 25747‐1‐AP] at 4°C overnight and FITC‐conjugated goat anti‐rabbit IgG [dilution 1:500; Yeasen, 33107ES60] at room temperature for 1 hour to identify monocytes/macrophages. The fluorescent dye 4′,6‐diamidino‐2‐phenylindole dihydrochloride (DAPI) (beyotime, C1005) was finally added to the sections to mark the nuclei. Images were collected using an Olympus IX71 fluorescence microscope.

### 2.13 Cellular experiment

RAW264.7 cells (murine macrophage cell line) were purchased form from the Cell Bank of the Chinese Academy of Sciences (Shanghai, China) and maintained in RPMI1640 (Gibco, Thermo Fisher Scientific) containing 10% (v/v) fetal bovine serum (FBS, Gibco, Thermo Fisher Scientific). The cells were cultured at 37°C in a humidified atmosphere with 5% CO_2_.

RAW264.7 cells were seeded in 6‐well tissue culture plate at a density of 5 × 10^5^ per well. After adherence, the cell were treated with 100 ng mL^−1^ lipopolysaccharides (LPS) for 12 hours and then exposed to LIPUS therapy. After LIPUS delivery, the cells were stored for 6 hours in the same medium before protein extraction. The expression of P‐caveolin‐1, P‐p38 MAPK, P‐ERK, IL‐6 and TNF‐α were measured by western blotting. The expression of P‐caveolin‐1 of RAW264.7 was also investigated by immunofluorescence.

Moreover, to verify whether the effect of LIPUS on RAW264.7 cells post‐LPS treatment was related to caveolin‐1, siRNA‐mediated knockdown of caveolin‐1 (Riobio technologies, Guangzhou, China) was performed. RAW264.7 cells were transfected with siCaveolin‐1 by using Lipofectamine^®^ 2000 reagent (Invitrogen, Carlsbad, USA) for 6 hours at 37°C, then treated with LPS and LIPUS subsequently, changes in protein expression of P‐p38 MAPK, P‐ERK, TNF‐α and IL‐6 were measured by western blotting.

### 2.14 Statistical methods

Data were expressed as means ± standard deviation (SD). Shapiro‐Wilk test was used for normal distribution. If distribution of the data was normal, outcomes were compared among groups using 1‐way ANOVA followed by the Dunnett multiple‐comparison test. If not, Wilcoxon's rank sum test should be applied instead. The Kaplan‐Meier method was applied to calculate survival rate. The differences in the pathological scores were evaluated using Krusal‐Wallis test. All the analyses were performed with SPSS 17.0 statistical software. A value of *P* < 0.05 was considered significant.

## 3 RESULTS

### 3.1 Low‐intensity pulsed ultrasound improved the general status of CVB3‐infected mice during the follow‐up of 14 days

**Figure 1:**
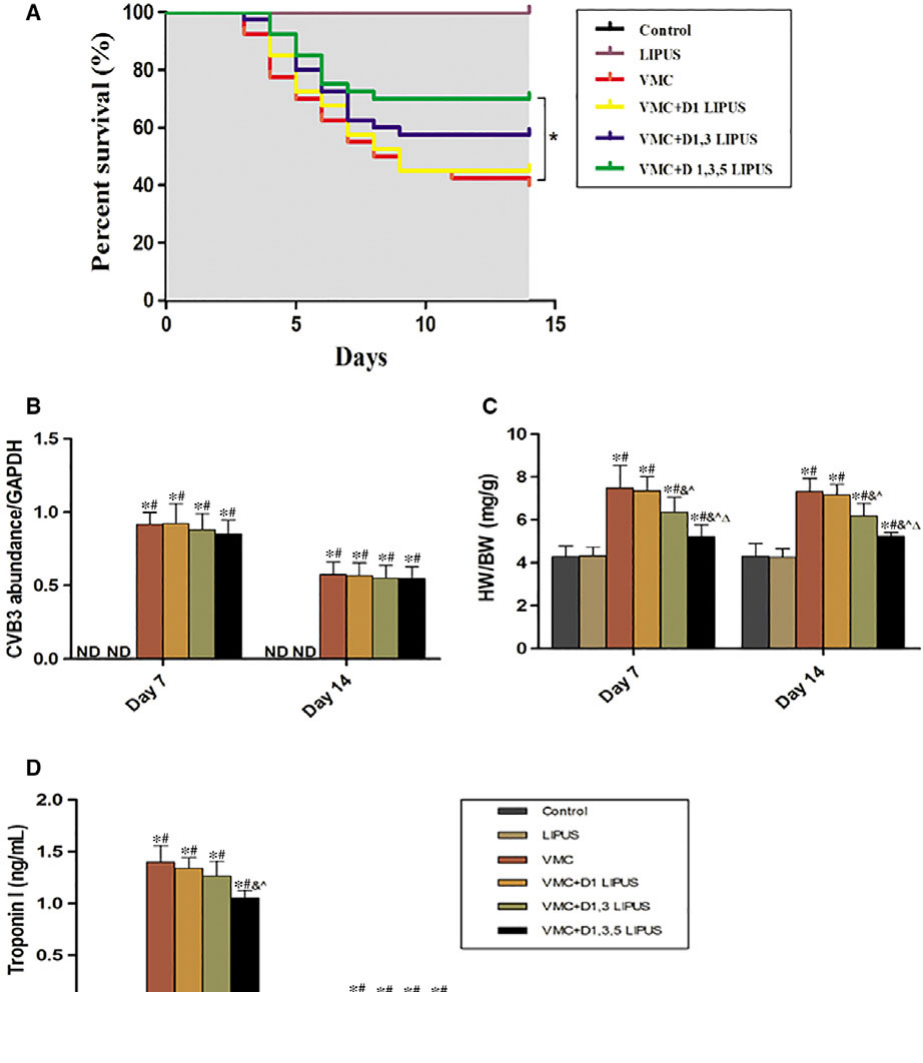
Day 1, 3, 5 LIPUS treatment improved survival, reduced heart weight/body weight (HW/BW) and decreased plasma Troponin I level of CVB3‐infected mice; however, it had no effect on the replication of CVB3 virus. A, The survival curve of each group. n = 40 in each group. *P < 0.05 vs VMC group. B, CVB3 replication on Day 7 and Day 14 from each group. n = 6 in each group. ND, not detected, *P < 0.05 vs Control group, ^#^ *P* < 0.05 vs LIPUS group. C, HW/BW on Day 7 and Day 14 from each group. n = 6 in each group. *P < 0.05 vs Control group, ^#^ *P* < 0.05 vs LIPUS group, ^&^ *P* < 0.05 vs VMC group, ^*P* < 0.05 vs VMC + D1 LIPUS group, ^Δ^ *P* < 0.05 vs VMC + D1, 3 LIPUS. D, Plasma Troponin I on Day 7 and Day 14 from each group. n = 6 in each group. *P < 0.05 vs Control group, ^#^ *P* < 0.05 vs LIPUS group, ^&^ *P* < 0.05 vs VMC group, ^*P* < 0.05 vs VMC + D1 LIPUS group, ^Δ^ *P* < 0.05 vs VMC + D1, 3 LIPUS

After CVB3 inoculation, mice exhibited signs of illness, including weight loss, anorexia, lethargy, back arching and irritability. The effect of LIPUS on viral myocarditis in terms of survival rate was first investigated. There was no animal death in Control group and LIPUS group during the follow‐up of 14 days. Compared with control and LIPUS group, VMC group had a poor survival with survival rate of 40% (16/40, *P* = 0.00). Despite no significant survival improvement observed in VMC + D1 LIPUS group (45%, 18/40, *P* = 0.588) and VMC + D1, 3 LIPUS group (57.5%, 23/40, *P* = 0.128) compared with VMC group, a significant increase of survival rate was observed in VMC + D1, 3, 5 LIPUS group (70%, 28/40, *P* = 0.013) (Figure 1A).

We then explored the effect of LIPUS treatment on CVB3‐RNA abundance in the myocardium of infected mice. There were no significant differences of CVB3 replication in myocardium between infected mice and infected mice receiving LIPUS treatment on days 7 and 14 (Figure 1B).

We also investigated the effect of LIPUS on heart weight/body weight (HW/BW) of mice induced viral myocarditis. On Day 7 and Day 14 after virus inoculation, there was an increased HW/BW detected in mice of VMC group (*P* < 0.05). VMC + D1 LIPUS group showed no obvious difference of HW/BW with VMC group (*P* > 0.05). Intriguingly, D1, 3 LIPUS and D1, 3, 5 LIPUS treatment significantly decreased the HW/BW of CVB3‐infected mice (*P* < 0.05). In addition, a more decreased HW/BW was observed on mice receiving D1, 3, 5 LIPUS treatment (*P* < 0.05) (Figure 1C).

Plasma Troponin I was a sensitive biomarker for detecting cardiac injury induced by acute viral myocarditis in clinic. We then explored whether LIPUS treatment mediated a reduction of Troponin I in CVB3‐infected mice. On Day 7, compared with Control group and LIPUS group, CVB3 infection induced an obvious elevation of Troponin I (*P* < 0.05). While D1 LIPUS and D1, 3 LIPUS treatment did not reduce the Troponin I level significantly (*P* > 0.05), D1, 3, 5 LIPUS treatment elicit an obvious reduction of Troponin I level (*P* < 0.05). On Day 14, the level of Troponin I declined remarkably in VMC group, there was no significant difference of Troponin I among VMC, VMC + D1 LIPUS, VMC + D1, 3 LIPUS and VMC + D1, 3, 5 LIPUS groups (*P* > 0.05) (Figure 1D).

### 3.2 Low‐intensity pulsed ultrasound improved the left ventricular function in CVB3‐infected mice

**Figure 2:**
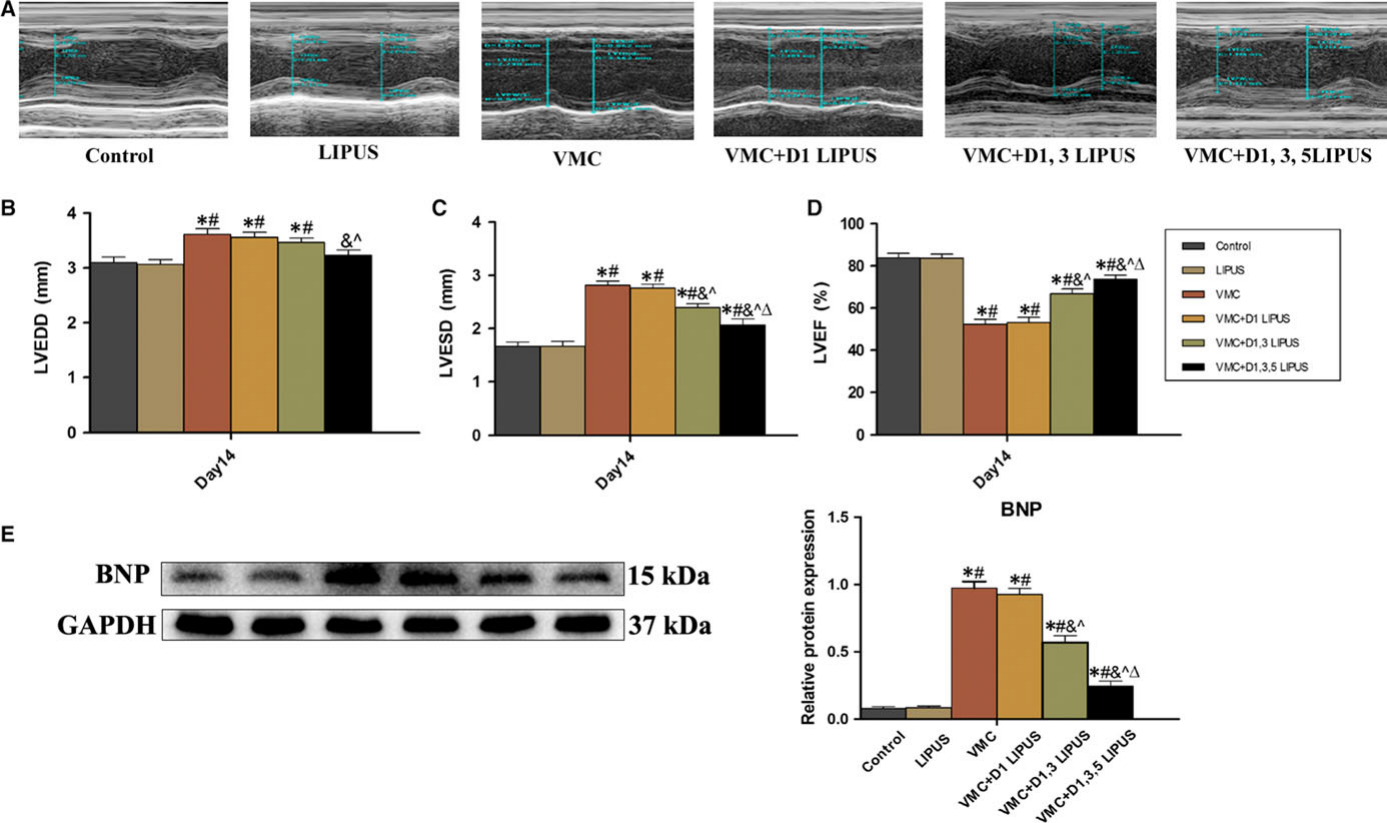
Transthoracic echocardiography and cardiac brain natriuretic peptide (BNP) revealed that Day 1, 3, 5 LIPUS treatment elicited a heart function improvement of CVB3‐infected mice. A, Representative echocardiography of mice from each group. B, Echocardiographic analysis of left ventricular end‐diastolic diameter (LVEDD) of mice from each group. n = 6 in each group. *P < 0.05 vs Control group, ^#^ *P* < 0.05 vs LIPUS group, ^&^ *P* < 0.05 vs VMC group, ^*P* < 0.05 vs VMC + D1 LIPUS group, ^Δ^ *P* < 0.05 vs VMC + D1, 3 LIPUS. C, Echocardiographic analysis of left ventricular end‐systolic diameter (LVESD) of mice from each group. n = 6 in each group. *P < 0.05 vs Control group, ^#^ *P* < 0.05 vs LIPUS group, ^&^ *P* < 0.05 vs VMC group, ^*P* < 0.05 vs VMC + D1 LIPUS group, ^Δ^ *P* < 0.05 vs VMC + D1, 3 LIPUS. D, Echocardiographic analysis of left ventricular eject fraction (LVEF) of mice from each group. n = 6 in each group. *P < 0.05 vs Control group, ^#^ *P* < 0.05 vs LIPUS group, ^&^ *P* < 0.05 vs VMC group, ^*P* < 0.05 vs VMC + D1 LIPUS group, ^Δ^ *P* < 0.05 vs VMC + D1, 3 LIPUS. E, Cardiac expression of BNP of mice from each group. n = 6 in each group. *P < 0.05 vs Control group, ^#^ *P* < 0.05 vs LIPUS group, ^&^ *P* < 0.05 vs VMC group, ^*P* < 0.05 vs VMC + D1 LIPUS group, ^Δ^ *P* < 0.05 vs VMC + D1, 3 LIPUS

On day 14, transthoracic echocardiography was utilized to evaluate the left ventricular function of mice from each group. Compared with mice in Control group and LIPUS group, mice in VMC group exhibited an obvious deteriorated heart function, manifesting as enlarged left ventricular end‐diastolic and end‐systolic diameter and declined left ventricular ejection function (*P *< 0.05). Mice in VMC + D1 LIPUS group showed no apparent differences with mice in VMC group regarding these echocardiographic parameters. Compared with mice in VMC group, mice receiving D1, 3 and D1, 3, 5 LIPUS treatment presented an significant improvement in heart function with an increased left ventricular ejection fraction (*P* < 0.05). In addition, a more improvement of heart function was observed in VMC + D1, 3, 5 LIPUS group (*P* < 0.05) (Figure 2A‐D).

The cardiac expression of brain natriuretic peptide (BNP) was also measured to monitor the heart function of mice from each group. On Day 14, Compared with Control group and LIPUS group, there was an increased expression of cardiac BNP in VMC group (*P* < 0.05). VMC + D1 LIPUS group exhibited no significant difference of BNP with VMC group. The BNP level was largely decreased in VMC + D1, 3 LIPUS and VMC + D1, 3, 5 LIPUS groups (*P* < 0.05) and a higher level of BNP decline was observed in VMC + D1, 3, 5 LIPUS group (*P* < 0.05) (Figure 2E).

### 3.3 LIPUS attenuated cardiac histopathological changes in CVB3‐infected mice

**Figure 3:**
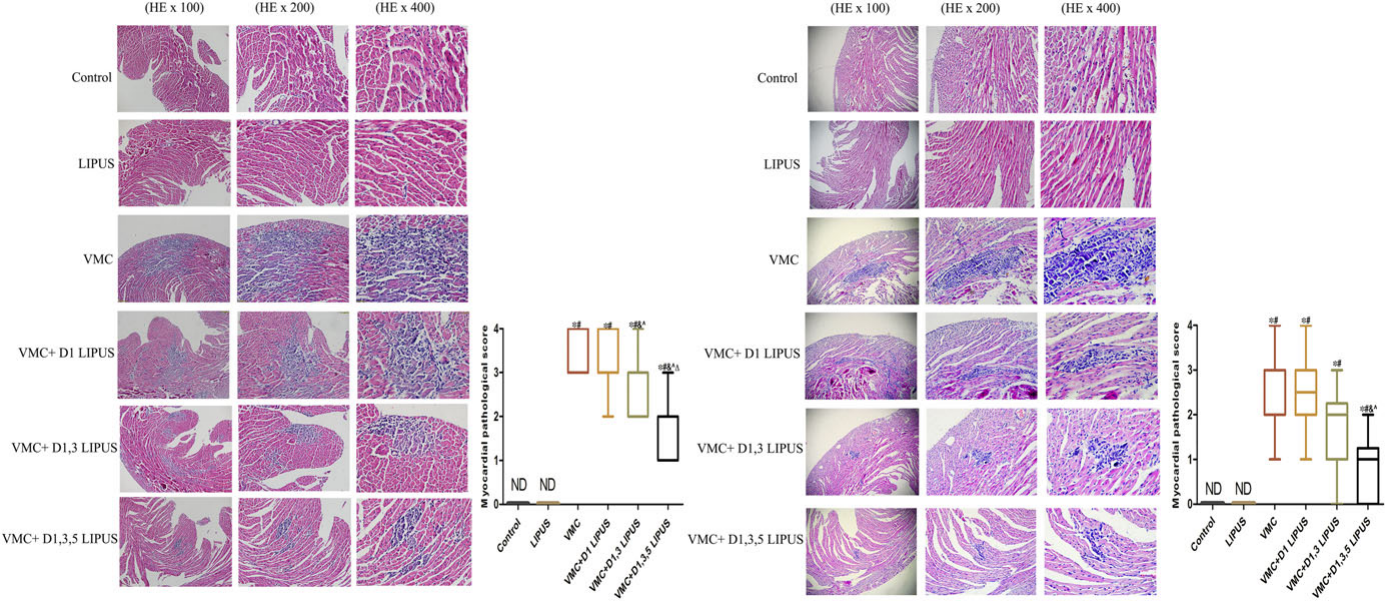
Day 1, 3, 5 LIPUS treatment attenuated the myocardial histopathological changes in CVB3‐infected mice. A, Myocardial histopathological changes of mice from each group on day 7 post‐virus inoculation. n = 6 in each group. *P < 0.05 vs Control group, ^#^ *P* < 0.05 vs LIPUS group, ^&^ *P* < 0.05 vs VMC group, ^*P* < 0.05 vs VMC + D1 LIPUS group, ^Δ^ *P* < 0.05 vs VMC + D1, 3 LIPUS. B, Myocardial histopathological changes of mice from each group on day 14 post‐virus inoculation. n = 6 in each group. *P < 0.05 vs Control group, ^#^ *P* < 0.05 vs LIPUS group, ^&^ *P* < 0.05 vs VMC group, ^*P* < 0.05 vs VMC + D1 LIPUS group, ^Δ^ *P* < 0.05 vs VMC + D1, 3 LIPUS

On day 7 and day 14, histopathological changes in heart tissue from each group were observed by HE staining. While normal tissue architecture was observed in Control group and LIPUS group, a pronounced inflammatory infiltration was observed both on day 7 and day 14 in VMC group. On day 7, although the gross histopathological characteristics did not differ between VMC group and VMC + D1 LIPUS group (*P* > 0.05), a significantly reduced inflammatory infiltration was observed in VMC + D1, 3 LIPUS group and VMC + D1, 3, 5 LIPUS group (*P *< 0.05), and a more obvious alleviation of cardiac inflammation was observed in VMC + D1, 3, 5 LIPUS group (*P* < 0.05) (Figure 3A). On day 14, the gross histopathological changes ameliorated slightly in each group, and compared with VMC group, a significant reduction of inflammatory infiltration was detected in VMC+D1, 3, 5 LIPUS group (*P* < 0.05) (Figure 3B).

### 3.4 LIPUS decreased the expression of inflammatory cytokine in heart tissue of CVB3‐infected mice

**Figure 4:**
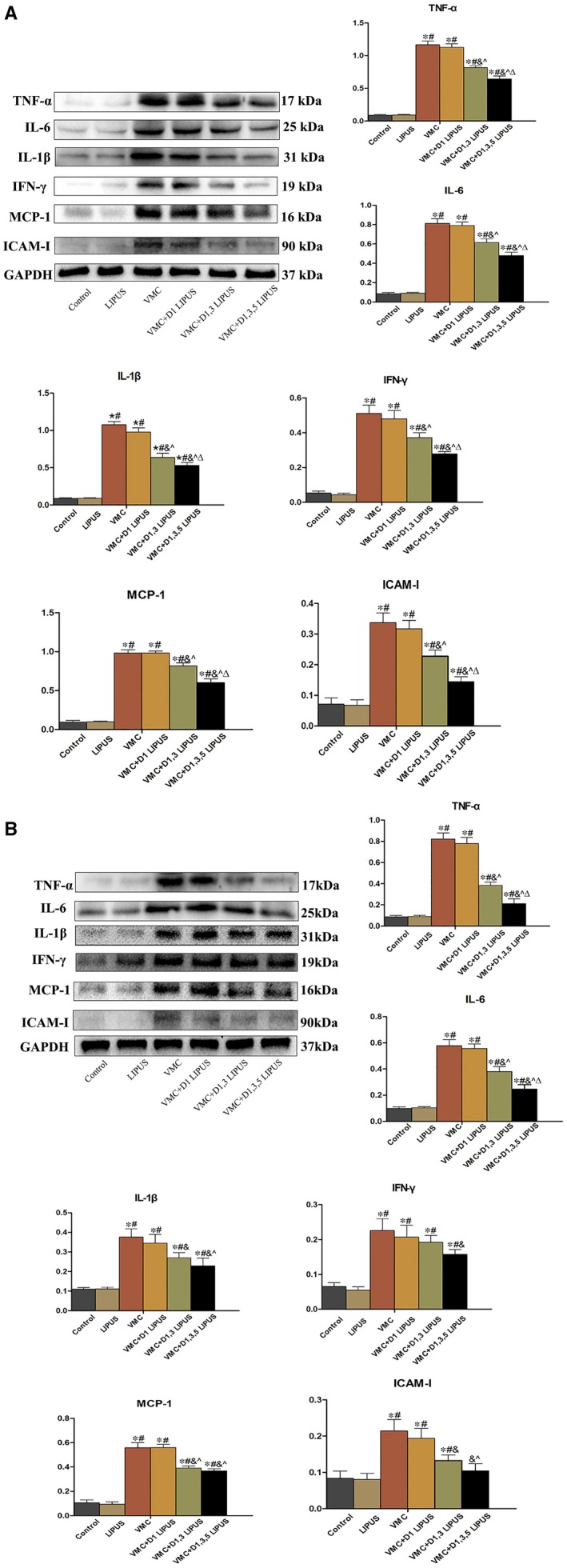
Day 1, 3, 5 LIPUS treatment declined the expression of pro‐inflammatory cytokines (TNF‐α, IL‐6, IL‐1β, IFN‐γ, ICAM‐1 and MCP‐1) in heart tissue of CVB‐infected mice. A, Pro‐inflammatory cytokines expression in heart tissue from each group on Day 7. N = 6 in each group. *P < 0.05 vs Control group, ^#^ *P* < 0.05 vs LIPUS group, ^&^ *P* < 0.05 vs VMC group, ^*P* < 0.05 vs VMC + D1 LIPUS group, ^Δ^ *P* < 0.05 vs VMC + D1, 3 LIPUS. B, A, Pro‐inflammatory cytokines expression in heart tissue from each group on Day 14. n = 6 in each group. *P < 0.05 vs Control group, ^#^ *P* < 0.05 vs LIPUS group, ^&^ *P* < 0.05 vs VMC group, ^*P* < 0.05 vs VMC + D1 LIPUS group, ^Δ^ *P* < 0.05 vs VMC + D1, 3 LIPUS

A series of inflammatory cytokines were measured in heart tissue by western blot to explore the effect on LIPUS on viral myocarditis. On day 7, compared with Control group and LIPUS group, there was an increased expression of pro‐inflammatory cytokines (TNF‐α, IL‐6, IL‐1β, IFN‐γ, ICAM‐1 and MCP‐1) in VMC group (*P* < 0.05). Though D1 LIPUS treatment did not lower the expression of pro‐inflammatory cytokines, D1, 3 LIPUS and D1, 3, 5 LIPUS treatment significantly decreased the cardiac expression of pro‐inflammatory cytokines (*P* < 0.05) (Figure 4A). Additionally, D1, 3, 5 LIPUS treatment reduced the expression of cytokines to a much larger degree. On day 14, the cardiac inflammation did not resolve completely, there was still a higher expression of pro‐inflammatory cytokines in CVB3‐infected mice (*P* < 0.05). Consistent with the observation on day 7, D1, 3, 5 LIPUS treatment delivered a significant benefit of pro‐inflammatory cytokines reduction on day 14 (*P* < 0.05) (Figure 4B).

### 3.5 LIPUS treatment increased the activation of caveolin‐1 and decreased the phosphorylation of p38 MAPK and ERK in heart tissue of CVB3‐infected mice

**Figure 5:**
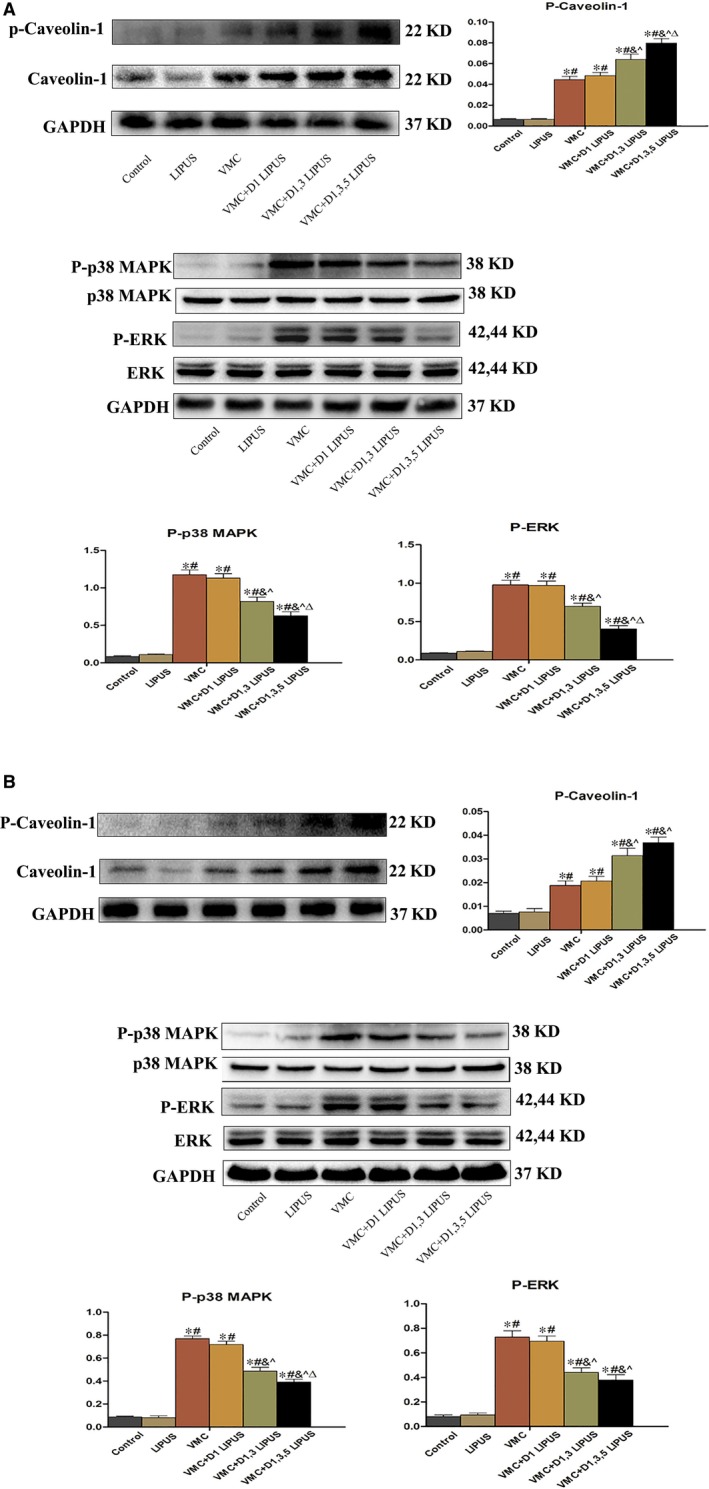
Day 1, 3, 5 LIPUS treatment led an increased activation of caveolin‐1 and a suppressed phosphorylation of p38 MAPK and ERK in heart tissue of CVB‐infected mice. A, Expression of P‐Caveolin‐1, p38MAPK and ERK in heart tissue from each group on Day 7. n = 6 in each group. *P < 0.05 vs Control group, ^#^ *P* < 0.05 vs LIPUS group, ^&^ *P* < 0.05 vs VMC group, ^*P* < 0.05 vs VMC + D1 LIPUS group, ^Δ^ *P* < 0.05 vs VMC + D1, 3 LIPUS. B, Expression of P‐Caveolin‐1, p38MAPK and ERK in heart tissue from each group on Day 14. n = 6 in each group. *P < 0.05 vs Control group, ^#^ *P* < 0.05 vs LIPUS group, ^&^ *P* < 0.05 vs VMC group, ^*P* < 0.05 vs VMC + D1 LIPUS group, ^Δ^ *P* < 0.05 vs VMC + D1, 3 LIPUS

As previous studies revealed that LIPUS treatment could elicit a mechanical stimulation of caveolin‐1, and MAPK signallings have been demonstrated critically involved in inflammatory response and regulated by caveolin‐1, we further investigated the effect of LIPUS on cardiac expression of caveolin‐1 and MAPK signallings in viral myocarditis. On day 7, compared with Control group and LIPUS group, there was an increased phosphorylation of caveolin‐1, p38 MAPK and ERK in VMC group (*P* < 0.05). Though the expression of P‐caveolin‐1, P‐p38 MAPK and P‐ERK did not differ between VMC group and VMC + D1 LIPUS group, D1, 3 LIPUS and D1, 3, 5 LIPUS treatment significantly increased the phosphorylation of caveolin‐1 and decreased the phosphorylation of p38MAPK and ERK (*P* < 0.05) (Figure 5A). Additionally, D1, 3, 5 LIPUS treatment increased P‐caveolin‐1 and decreased P‐p38 MAPK and P‐ERK to a much larger extent. On day 14, there was still a higher expression of P‐caveolin‐1, P‐p38 MAPK and P‐ERK in VMC group compared with Control and LIPUS group (*P* < 0.05). Besides, VMC + D1, 3, 5 LIPUS group still exhibited a much higher expression of P‐caveolin‐1 and lower expression of P‐p38 MAPK and P‐ERK on day 14 (*P* < 0.05) (Figure 5B).

### 3.6 LIPUS treatment suppressed the infiltration of CD68^+^ cell in heart tissue of CVB3‐infected mice

**Figure 6:**
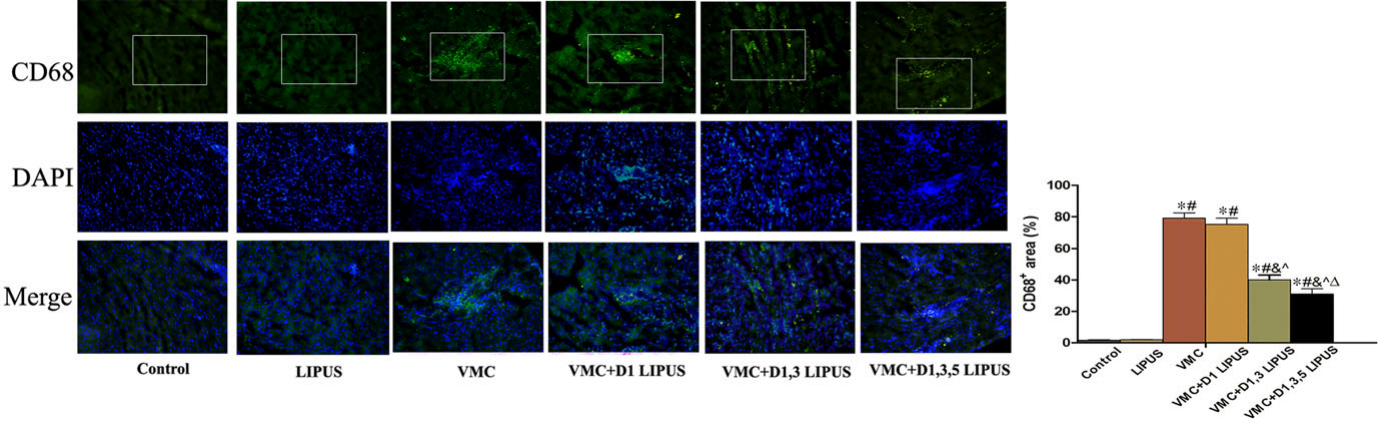
Day 1, 3, 5 LIPUS treatment inhibited macrophages infiltration in heart tissue of CVB3‐infected mice. Representative immunofluorescence staining of CD68^+^ cells in heart tissue from each group (magnification ×200). n = 6 in each group. *P < 0.05 vs Control group, ^#^ *P* < 0.05 vs LIPUS group, ^&^ *P* < 0.05 vs VMC group, ^*P* < 0.05 vs VMC + D1 LIPUS group, ^Δ^ *P* < 0.05 vs VMC + D1, 3 LIPUS

During the inflammatory process of acute viral myocarditis, macrophages acted as the first‐line immune cells and participated in the initiation and promotion of aggressive immunological response. Given a more pronounced inflammatory response and cardiac injury was observed on day 7, we further evaluated the infiltration of macrophages (marked by CD68^+^) in heart tissue on day 7 by immunofluorescence staining. Compared with mice in Control group and LIPUS group, a large amount of macrophages were detected in the heart tissue of mice fromVMC group (*P* < 0.05). Though D1 LIPUS treatment did not inhibit the infiltration of macrophages (*P* > 0.05), D1, 3 LIPUS and D1, 3, 5 LIPUS treatment significantly decreased the amount of macrophages (*P* < 0.05). In addition, a higher suppression of macrophages was observed in VMC+D1, 3, 5 LIPUS treatment (*P* < 0.05) (Figure 6). Based on this phenomenon, we confirmed that during acute viral myocarditis, macrophages were critically influenced by LIPUS treatment. Thus, we concentrated on macrophage in vitro to elucidate the possible mechanisms of LIPUS treatment on CVB3‐induced myocarditis.

### 3.7 LIPUS treatment alleviated LPS‐induced inflammatory response on RAW264.7 by increasing the activation of caveolin‐1 and suppressing the phosphorylation of p38 MAPK and ERK

**Figure 7:**
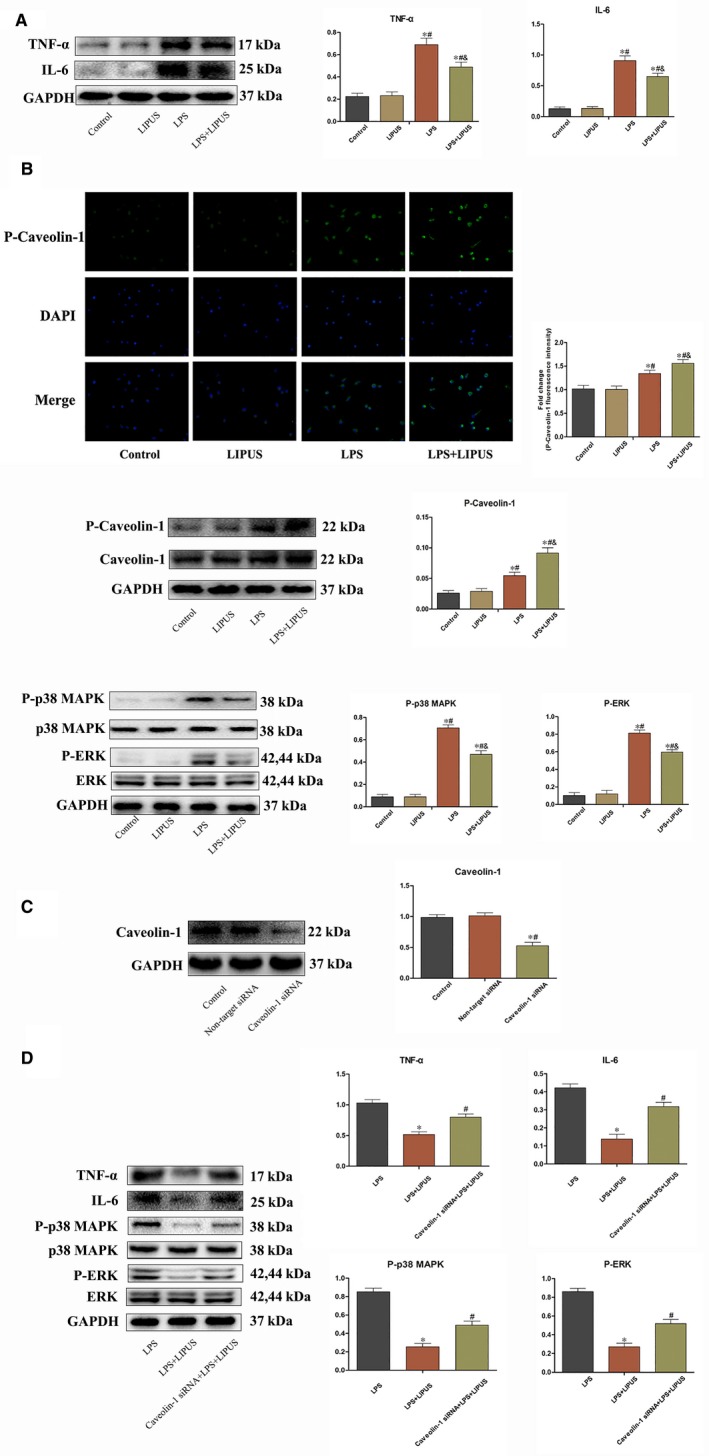
LIPUS treatment alleviated the LPS‐induced inflammatory response on RAW264.7 by increasing the activation of caveolin‐1 and suppressing the phosphorylation of p38 MAPK and ERK. A, LIPUS inhibited the pro‐inflammatory cytokines (TNF‐α and IL‐6) expression of RAW264.7 treated with LPS. n = 6 in each group. *P < 0.05 vs Control group, ^#^ *P* < 0.05 vs LIPUS group, ^&^P < 0.05 vs LPS group. B, LIPUS increased P‐caveolin‐1 and suppressed P‐p38 MAPK and P‐ERK of RAW264.7 treated with LPS. Immunofluorescence staining of P‐caveolin‐1 in control, LIPUS, LPS and LPS + LIPUS cells (magnification ×400). *P < 0.05 vs Control group, ^#^ *P* < 0.05 vs LIPUS group, ^&^ *P* < 0.05 vs LPS group. P‐caveolin‐1, P‐p38 MAPK and P‐ERK expression in control, LIPUS, LPS and LPS + LIPUS treatment cells. n = 6 in each group. *P < 0.05 vs Control group, ^#^ *P* < 0.05 vs LIPUS group, ^&^ *P* < 0.05 vs LPS group. C, Transfection of RAW264.7 with caveolin‐1 siRNA down‐regulated the expression of caveolin‐1. n = 6 in each group. *P < 0.05 vs Control group, ^#^ *P* < 0.05 vs RAW264.7 transfected with non‐target siRNA. D, Transfection of RAW264.7 with caveolin‐1 siRNA blunted the inhibitory effect of LIPUS on pro‐inflammatory cytokines (TNF‐α and IL‐6) and P‐p38 MAPK and P‐ERK. n = 6 in each group. *P < 0.05 vs LPS group, ^#^ *P* < 0.05 vs LPS + LIPUS group

Given the catastrophic cardiac injury during acute viral myocarditis was caused by enlarged innate immunological activity, but not the CVB3 itself. Thus, in vitro study, the LPS was utilized on RAW264.7 to mimic the overactive immunological response during acute viral myocarditis. Compared with Control and LIPUS group, LPS treatment increased the expression of TNF‐α and IL‐6 (*P* < 0.05), however, LIPUS treatment decreased LPS‐induced elevation of pro‐inflammatory cytokines (*P* < 0.05) (Figure 7A). LPS‐treated RAW264.7 exhibited an increased expression of P‐caveolin‐1, P‐p38 MAPK and P‐ERK (*P* < 0.05), however, LIPUS treatment post‐LPS inoculation led a more increase of P‐caveolin‐1 and higher suppression of P‐p38 MAPK and P‐ERK (*P* < 0.05) (Figure 7B). The relationship between caveolin‐1 and MAPK signallings under LIPUS treatment was further explored. After transfected with caveolin‐1 siRNA, the expression of caveolin‐1 in RAW264.7 was remarkably downregulated (*P* < 0.05) (Figure 7C). With the knock‐down of caveolin‐1, the inhibitory effect of LIPUS on P‐p38 MAPK, P‐ERK and pro‐inflammatory cytokines (TNF‐α and IL‐6) was significantly blunted (*P* < 0.05) (Figure 7D).

## 4 DISCUSSION

In this study, we first revealed that low‐intensity pulsed ultrasound treatment could alleviate the drastic cardiac inflammatory response elicited by CVB3 infection and benefit the disease course of acute viral myocarditis. Based on in vivo and vitro explorations, we proposed that mechanical activation of caveolin‐1 and further regulation of MAPK signallings were key mechanisms involved in the beneficial effect of LIPUS treatment.

Viral myocarditis was a disease marked by inflammation and damage of the heart muscle induced by virus infection. It was believed that 5% to 20% of all cases of sudden death in young adults were due to viral myocarditis. After virus invading into the body, in the early phase (1‐3 days), virus itself caused a direct and immediate cardiac injury by infecting cardiomyocytes and inducing local immune response. During the following 4‐10 days, the CVB3‐mediated cardiac injury was augmented by an enlarged innate immunological response, which was characterized as overactive inflammatory cells attacking normal myocardium and resulted in a large amount of myocardium loss, contributing to the occurrence of acute heart failure and even sudden death in clinic. Though there was no effective therapy for viral myocarditis currently, limiting the aggressive inflammatory response during the acute viral myocarditis may be a potential and effective treatment.^1^, ^2^, ^3^, ^4^, ^5^

Low‐intensity pulsed ultrasound was a form of mechanical energy delivered via a special device and resulted in activation of mechanotransduction system of various cells.^19^ Recently, LIPUS has been proven to ameliorate inflammatory activity in a series of animal experiments. Tatsuya Nakamura et al. once reported that LIPUS treatment alleviated synovitis in the knee joints of animal models for rheumatoid arthritis.^20^ In acute muscle tissue injury, LIPUS treatment led to reductions in the number of neutrophils and M1 macrophages, and consequently attenuated tissue inflammation and fibrosis.^11^ In TAC‐induced heart failure mice model, LIPUS treatment were revealed ameliorating perivascular fibrosis and myocardial ischaemia and suppressing macrophage infiltration.^14^ In this study, we found that LIPUS treatment on day 1, 3 and 5 after CVB3 inoculation remarkably increased animal survival, improved heart function, reduced cardiac histopathological lesions and declined a series of pro‐inflammatory cytokines. All these observations indicated that LIPUS possessed a potent of inflammatory regulation during acute viral myocarditis.

The underlying mechanism of LIPUS on viral myocarditis was further explored. Tomohiko Shindo et al. reported that after acute myocardial infarction, LIPUS treatment up‐regulated the expression of vascular endothelial growth factor and endothelial nitric oxide synthase in the infarcted area, leading to enhanced angiogenesis and attenuated left ventricular remodelling. However, in caveolin‐1‐deficient mice, the beneficial effects of LIPUS on post‐myocardial infarction left ventricular remodelling were absent.^13^ Their results indicated that caveolin‐1 played a key role in LIPUS treatment. In our study, we confirmed that LIPUS treatment alleviated the aggressive inflammatory response of acute viral myocarditis on mice and ameliorated the LPS‐induced inflammation on RAW264.7. In vivo, LIPUS treatment on day 1, 3, 5 post‐virus inoculation resulted in an increased activation of caveolin‐1. In vitro, LIPUS treatment also increased the expression of P‐caveolin‐1 of LPS‐treated RAW264.7, however, transfecting RAW264.7 with caveolin‐1siRNA blunted the beneficial effect of LIPUS. All of these phenomena observed in our study suggested that the beneficial effect of LIPUS on acute viral myocarditis was realized via the mechanical interaction between LIPUS energy and caveolin‐1.

The downstream intracellular signallings under LIPUS treatment were also investigated. Previous studies revealed that p38 MAPK and ERK were classical cellular signallings influenced by LIPUS treatment under inflammatory situations.^8^, ^21^, ^22^ Zhou et al. reported LIPUS treatment influenced the phagocytosis of macrophages via p38 MAPK and ERK signallings.^21^ Nakao et al demonstrated that LIPUS affected the inflammatory responses of osteoblasts to LPS, where LPS‐induced production of inflammatory cytokines and phosphorylation of ERK and p38MAPK were decreased by LIPUS treatment.^8^ In addition, recent studies indicated that p38 MAPK and ERK signallings played pivotal roles in the pathogenesis of viral myocarditis, and suppressing these signallings led to an inhibition of cardiac injury and benefited the recovery from viral myocarditis.^23^, ^24^, ^25^ In our study, we also found that p38 MAPK and ERK signallings were activated during viral myocarditis, while LIPUS treatment suppressed these signallings and attenuated the excessive inflammatory response during the disease course. Intriguingly, some studies showed that MAPK signallings could be further activated after receiving mechanical stimulation from LIPUS, which were contrary to our findings.^13^, ^26^ It was noteworthy to mention that these studies focused on the effect of LIPUS treatment on endothelial function and angiogenesis post‐ischaemia, while we investigated LIPUS in acute viral myocarditis. We thought that the opposite expression of MAPK signallings under LIPUS treatment may be due to different pathogenesis of different diseases. In addition, MAPK signallings were involved in several negative feedback loops and negatively regulated by members from dual‐specificity phosphatase (DUSP) family. DUSP genes induced by MAPKs activation could in turn dramatically inhibit the activation of MAPKs.^27^ The negative feedback regulation of MAPKs may further explain the discrepancy between our studies and others.

Whether there was a relationship between caveolin‐1 and MAPK signallings under LIPUS treatment was investigated by transfecting RAW264.7 with caveolin‐1siRNA. It has been reported that Caveolin‐1 regulated inflammatory response through MAPK signallings.^15^, ^16^, ^17^, ^18^ Elena Tourkina et al. previously reported the monocytes and neutrophils isolated from scleroderma patients contained less caveolin‐1 and more activated p38 MAPK and ERK, which was responsible for more leukocytes accumulation in the lung and much severer lung fibrosis. Restoring caveolin‐1 by caveolin‐1 scaffolding domain peptide treatment reversed p38 MAPK and ERK hyperactivation and lung lesions.^18^ In our study, LIPUS treatment increased the activation of caveolin‐1 and decreased the phosphorylation of p38 MAPK and ERK in vivo and in vitro. Besides, in vitro study, by transfecting RAW264.7 with Caveolin‐1 siRNA, LIPUS‐induced suppression of p38 MAPK and ERK signallings was abolished. Taken together, we proposed that LIPUS treatment attenuated CVB3‐induced myocardial inflammatory response via activation of caveolin‐1 and regulation of p38MAP and ERK signallings.

Several limitations should be mentioned for this study. First, as LIPUS treatment was performed within the first 5 days after virus inoculation, the effect of a relative longer course of LIPUS treatment on viral myocarditis was not investigated. However, based on our study, we confirmed the cardioprotective effect of LIPUS treatment given in the early phase of viral myocarditis. Second, we have not investigated LIPUS treatment on caveolin‐1 knochout (KO) mice. Though caveolin‐1 KO mice were not utilized in vivo study to testify the LIPUS effect on viral myocarditis, caveolin‐1 knockdown by small interfering RNA was performed in vitro to clarify the mechanism of LIPUS treatment. In addition, it has been reported that caveolin‐1 KO mice exhibited defected innate immunity and overactive inflammatory activity in response to various stress.^28^, ^29^ Thus, the beneficial effects of LIPUS on viral myocarditis might be blunted in caveolin‐1 KO mice. Third, the enlarged immunological response during the acute viral myocarditis was mimicked by LPS treatment on RAW264.7 in vitro but not by CVB3 infection. As an essential innate immune cell component, cardiac macrophages began to enrich at as early as day 3 but not immediately post‐CVB3 infection. Conditioned by the infected cardiac microenvironment but not CVB3, infiltrating macrophages initiated and promoted cardiac inflammation by secreting a series of pro‐inflammatory cytokines and further dictating the subsequent adaptive immunity. It was worth mentioning that the virus itself only caused local immune response with limited cardiac injury, whereas, the character took responsibility for the large amount of myocardium loss and poor prognosis during viral myocarditis was the overactive immunological activity. From this standpoint, we thought that LPS was more reasonable than CVB3 to simulate the overactive immunological response. Fourth, in this study, we only investigated LIPUS treatment on macrophage in vitro, leaving its effect on other cells not explored. A wide range of cardiac resident cells (cardiomyocytes, endothelial cells, fibroblasts) were also induced inflammation by CVB3 infection and assisted in shaping the inflammatory response during viral myocarditis,^30^ their inflammatory response may be also influenced by LIPUS treatment. However, as we aimed to investigate the LIPUS treatment on the aggressive immunological response during acute viral myocarditis, which was initiated and gone through by macrophages, thus we selected macrophages rather than other cells to do our explorations.

## 5 CONCLUSION

In this study, we revealed that the LIPUS therapy attenuated the inflammatory response and improved the survival of viral myocarditis, where activation of caveolin‐1 and regulation of MAPK signalling pathways were key mechanisms involved. Our study provided a new, promising and noninvasive strategy for the treatment of viral myocarditis.

## CONFLICT OF INTEREST

The authors declare that they have no conflict of interest.

## References

[1] Yajima T , Knowlton KU . Viral myocarditis: from the perspective of the virus. Circulation. 2009;119:2615‐2624. 19451363 10.1161/CIRCULATIONAHA.108.766022

[2] Yamada T , Matsumori A , Sasayama S . Therapeutic effect of anti‐tumor necrosis factor‐alpha antibody on the murine model of viral myocarditis induced by encephalomyocarditis virus. Circulation. 1994;89:846‐851. 8313574 10.1161/01.cir.89.2.846

[3] Nishio R , Matsumori A , Shioi T , Ishida H , Sasayama S . Treatment of experimental viral myocarditis with interleukin‐10. Circulation. 1999;100:1102‐1108. 10477536 10.1161/01.cir.100.10.1102

[4] Mann DL . Tumor necrosis factor and viral myocarditis: the fine line between innate and inappropriate immune responses in the heart. Circulation. 2001;103:626‐629. 11156870 10.1161/01.cir.103.5.626

[5] Marchant DJ , McManus BM . Regulating viral myocarditis: allografted regulatory T cells decrease immune infiltration and viral load. Circulation. 2010;121:2609‐2611. 20529996 10.1161/CIRCULATIONAHA.110.960054

[6] Jia L , Wang Y , Chen J , Chen W . Efficacy of focused low‐intensity pulsed ultrasound therapy for the management of knee osteoarthritis: a randomized, double blind, placebo‐controlled trial. Sci Rep. 2016;6:35453. 27748432 10.1038/srep35453 PMC5066246

[7] Saito R , Nagase T , Tateishi T , et al. Outcome of low‐intensity pulsed ultrasound (LIPUS) for opening wedge high tibial osteotomy. J Orthop Trauma. 2017;31:S3. 10.1097/01.bot.0000520893.61975.91 28632664

[8] Nakao J , Fujii Y , Kusuyama J , et al. Low‐intensity pulsed ultrasound (LIPUS) inhibits LPS‐induced inflammatory responses of osteoblasts through TLR4‐MyD88 dissociation. Bone. 2014;58:17‐25. 24091132 10.1016/j.bone.2013.09.018

[9] Jia L , Chen J , Wang Y , Zhang Y , Chen W . Focused low‐intensity pulsed ultrasound affects extracellular matrix degradation via decreasing chondrocyte apoptosis and inflammatory mediators in a surgically induced osteoarthritic rabbit model. Ultrasound Med Biol. 2016;42:208‐219. 26403700 10.1016/j.ultrasmedbio.2015.08.010

[10] Nagao M , Tanabe N , Manaka S , et al. Low‐intensity pulsed ultrasound inhibits lipopolysaccharide‐induced IL‐6 and RANKL expression in osteoblasts. J Oral Sci. 2017;59:303‐309. 28637991 10.2334/josnusd.16-0624

[11] da Silva Junior EM , Mesquita‐Ferrari RA , Franca CM , Andreo L , Bussadori SK , Fernandes KPS . Modulating effect of low intensity pulsed ultrasound on the phenotype of inflammatory cells. Biomed Pharmacother. 2017;96:1147‐1153. 29191696 10.1016/j.biopha.2017.11.108

[12] Hanawa K , Ito K , Aizawa K , et al. Low‐intensity pulsed ultrasound induces angiogenesis and ameliorates left ventricular dysfunction in a porcine model of chronic myocardial ischemia. PLoS ONE. 2014;9:e104863. 25111309 10.1371/journal.pone.0104863 PMC4128732

[13] Shindo T , Ito K , Ogata T , et al. Low‐intensity pulsed ultrasound enhances angiogenesis and ameliorates left ventricular dysfunction in a mouse model of acute myocardial infarction. Arterioscler Thromb Vasc Biol. 2016;36:1220‐1229. 27079882 10.1161/ATVBAHA.115.306477

[14] Ogata T , Ito K , Shindo T , et al. Low‐intensity pulsed ultrasound enhances angiogenesis and ameliorates contractile dysfunction of pressure‐overloaded heart in mice. PLoS ONE. 2017;12:e0185555. 28957396 10.1371/journal.pone.0185555 PMC5619801

[15] Wang XM , Kim HP , Song R , Choi AM . Caveolin‐1 confers antiinflammatory effects in murine macrophages via the MKK3/p38 MAPK pathway. Am J Respir Cell Mol Biol. 2006;34:434‐442. 16357362 10.1165/rcmb.2005-0376OC PMC2644205

[16] Wang J , Chen H , Cao P , et al. Inflammatory cytokines induce caveolin‐1/beta‐catenin signalling in rat nucleus pulposus cell apoptosis through the p38 MAPK pathway. Cell Prolif. 2016;49:362‐372. 27125453 10.1111/cpr.12254 PMC6496863

[17] Liu Z , Wang L , Dong Z , et al. Heparin inhibits lipopolysaccharide‐induced inflammation via inducing caveolin‐1 and activating the p38/mitogen‐activated protein kinase pathway in murine peritoneal macrophages. Mol Med Rep. 2015;12:3895‐3901. 25998703 10.3892/mmr.2015.3807

[18] Tourkina E , Richard M , Oates J , et al. Caveolin‐1 regulates leucocyte behaviour in fibrotic lung disease. Ann Rheum Dis. 2010;69:1220‐1226. 20410070 10.1136/ard.2009.117580 PMC2907085

[19] Izadifar Z , Babyn P , Chapman D . Mechanical and biological effects of ultrasound: a review of present knowledge. Ultrasound Med Biol. 2017;43:1085‐1104. 28342566 10.1016/j.ultrasmedbio.2017.01.023

[20] Nakamura T , Fujihara S , Yamamoto‐Nagata K , Katsura T , Inubushi T , Tanaka E . Low‐intensity pulsed ultrasound reduces the inflammatory activity of synovitis. Ann Biomed Eng. 2011;39:2964‐2971. 21938555 10.1007/s10439-011-0408-0

[21] Zhou S , Bachem MG , Seufferlein T , Li Y , Gross HJ , Schmelz A . Low intensity pulsed ultrasound accelerates macrophage phagocytosis by a pathway that requires actin polymerization, Rho, and Src/MAPKs activity. Cell Signal. 2008;20:695‐704. 18207700 10.1016/j.cellsig.2007.12.005

[22] Zhao X , Zhao G , Shi Z , et al. Low‐intensity pulsed ultrasound (LIPUS) prevents periprosthetic inflammatory loosening through FBXL2‐TRAF6 ubiquitination pathway. Sci Rep. 2017;7:45779. 28378753 10.1038/srep45779 PMC5381120

[23] Niu L , Li C , Wang Z , Xu H , An X . Effects of the MAPK pathway and the expression of CAR in a murine model of viral myocarditis. Exp Ther Med. 2017;13:230‐234. 28123495 10.3892/etm.2016.3909 PMC5244858

[24] Yue‐Chun L , Guang‐Yi C , Li‐Sha G , et al. The protective effects of ivabradine in preventing progression from viral myocarditis to dilated cardiomyopathy. Front Pharmacol. 2016;7:408. 27847478 10.3389/fphar.2016.00408 PMC5088506

[25] Xu HF , Ding YJ , Zhang ZX , et al. MicroRNA21 regulation of the progression of viral myocarditis to dilated cardiomyopathy. Mol Med Rep. 2014;10:161‐168. 24804616 10.3892/mmr.2014.2205

[26] Lionetti V , Fittipaldi A , Agostini S , Giacca M , Recchia FA , Picano E . Enhanced caveolae‐mediated endocytosis by diagnostic ultrasound in vitro. Ultrasound Med Biol. 2009;35:136‐143. 18950933 10.1016/j.ultrasmedbio.2008.07.011

[27] Huang CY , Tan TH . DUSPs, to MAP kinases and beyond. Cell Biosci. 2012;2:24. 22769588 10.1186/2045-3701-2-24 PMC3406950

[28] Chen Z , Nie S , Qu M , et al. The autophagic degradation of Cav‐1 contributes to PA‐induced apoptosis and inflammation of astrocytes. Cell Death Dis. 2018;9:771. 29991726 10.1038/s41419-018-0795-3 PMC6039485

[29] Yuan K , Huang C , Fox J , et al. Elevated inflammatory response in caveolin‐1‐deficient mice with Pseudomonas aeruginosa infection is mediated by STAT3 protein and nuclear factor kappaB (NF‐kappaB). J Biol Chem. 2011;286:21814‐21825. 21515682 10.1074/jbc.M111.237628 PMC3122236

[30] Woudstra L , Juffermans LJM , van Rossum AC , Niessen HWM , Krijnen PAJ . Infectious myocarditis: the role of the cardiac vasculature. Heart Fail Rev. 2018;23:583‐595. 29536322 10.1007/s10741-018-9688-x PMC6010496

