# Supplementary material for: Low‐intensity pulsed ultrasound attenuates cardiac inflammation of CVB3‐induced viral myocarditis via regulation of caveolin‐1 and MAPK pathways
Source: J Cell Mol Med. 2018 Dec 27;23(3):1963–75. doi: 10.1111/jcmm.14098 (PMC6378187; doi:10.1111/jcmm.14098)

The effect of LIPUS on RAW264.7 has been investigated. We first investigated LIPUS effect on RAW264. 7 in the range of (0-180 mW/cm^2^) in terms of cell viability. We found that LIPUS treatment in the range of 0-140mW/cm^2^ had no any obvious inﬂuence on the cell viability of RAW 264.7. A slight but not significant reduction of cell viability was observed in ISATA of 180 mW/cm^2^ (Supplementary Fig 1).

**
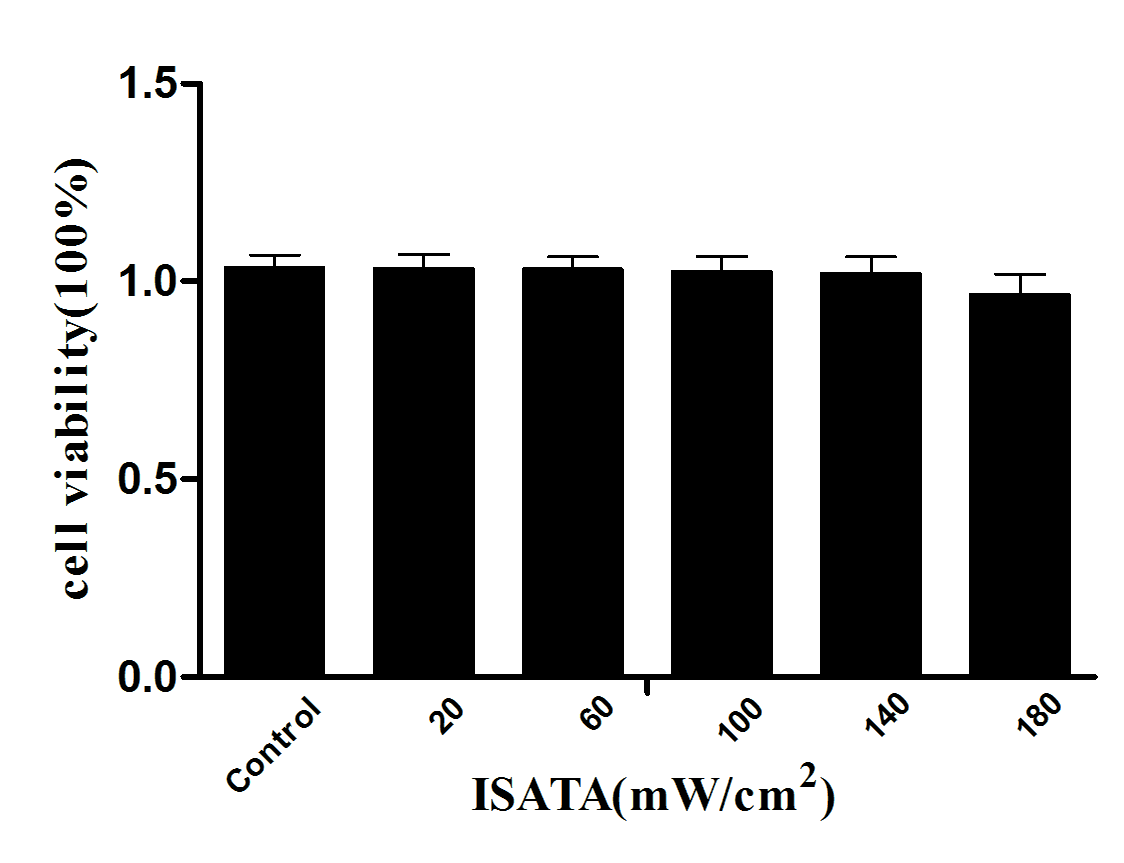
**

We then focused on whether LIPUS in the range of 0-140 mW/cm^2^ had an effect on LPS-treated RAW264.7 in terms of inflammatory response. We found that compared with LPS-treated RAW264.7, LIPUS of ISATA 100 mW/cm^2^ significantly decreased the expression of pro-inflammatory cytokines, while LIPUS of 140 mW showed no more inhibitory effect of pro-inflammatory cytokines than 100mW/cm^2^ (Supplementary Fig 2).


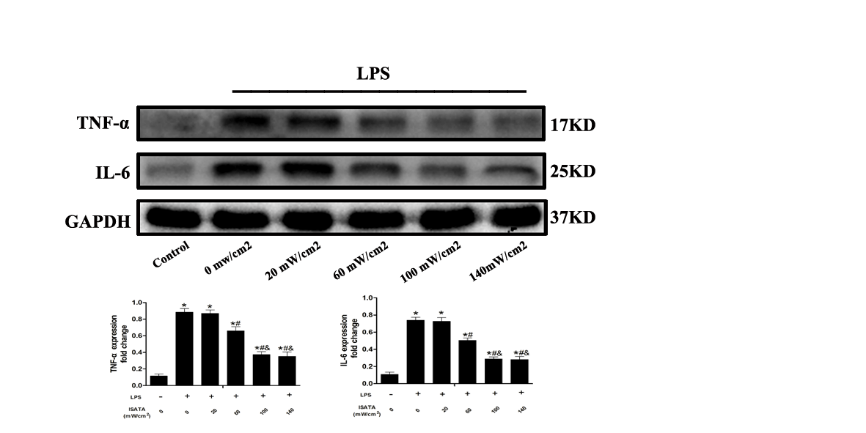

Supplement: Supplementary file 1 [file JCMM-23-1963-s001.docx]
